# New Zealand adolescents’ cellphone and cordless phone user-habits: are they at increased risk of brain tumours already? A cross-sectional study

**DOI:** 10.1186/1476-069X-12-5

**Published:** 2013-01-10

**Authors:** Mary Redmayne

**Affiliations:** 1 School of Geography, Environment and Earth Sciences, Science Faculty, Victoria University of Wellington, Wellington, New Zealand

**Keywords:** Mobile phone, Cellphone, Adolescents, Risk, Habits, Brain tumour

## Abstract

**Background:**

Cellphone and cordless phone use is very prevalent among early adolescents, but the extent and types of use is not well documented. This paper explores how, and to what extent, New Zealand adolescents are typically using and exposed to active cellphones and cordless phones, and considers implications of this in relation to brain tumour risk, with reference to current research findings.

**Methods:**

This cross-sectional study recruited 373 Year 7 and 8 school students with a mean age of 12.3 years (range 10.3-13.7 years) from the Wellington region of New Zealand. Participants completed a questionnaire and measured their normal body-to-phone texting distances. Main exposure-metrics included self-reported time spent with an active cellphone close to the body, estimated time and number of calls on both phone types, estimated and actual extent of SMS text-messaging, cellphone functions used and people texted. Statistical analyses used Pearson Chi^2^ tests and Pearson’s correlation coefficient (r). Analyses were undertaken using SPSS version 19.0.

**Results:**

Both cellphones and cordless phones were used by approximately 90% of students. A third of participants had already used a cordless phone for ≥ 7 years. In 4 years from the survey to mid-2013, the cordless phone use of 6% of participants would equal that of the highest Interphone decile (≥ 1640 hours), at the surveyed rate of use. High cellphone use was related to cellphone location at night, being woken regularly, and being tired at school. More than a third of parents thought cellphones carried a moderate-to-high health risk for their child.

**Conclusions:**

While cellphones were very popular for entertainment and social interaction via texting, cordless phones were most popular for calls. If their use continued at the reported rate, many would be at increased risk of specific brain tumours by their mid-teens, based on findings of the Interphone and Hardell-group studies.

## Background

Today’s young adolescents have grown up with cordless phones and cellphones in their homes, and commonly with old cellphones available to use as toys at home and in pre-schools. This equipment is therefore an integral part of their everyday lives. In the US, SMS (texting) now dominates young adolescents’ communication choices, and the use of cellphones, as a way to develop and maintain social interactions, is growing [1].

Studies to assess young people’s telephone user-habits have generally focused on cellphones. A German study found 34.7% of mostly 9–10 year-olds owned a cellphone by late 2002 [2]. The following year 45% of English students were found to own one [3]. By 2005, 76% of Hungarian 9–12 year-olds were reported owning a cellphone [4]. That year, 77% of Australian 11–13 year-olds had their own [5] and a Swedish group reported that ownership among students aged 7–14 grew from 7.3% in 7 year-olds, 57.8% aged 10 and 95% aged 14 [6]. In early 2007, 96.5% of Spanish students aged 13–20 years owned their own cellphone [7]. These studies demonstrate both increasing uptake over those years as well as increasing ownership with age. Extensive use was commonly associated with being female [4,6,7].

Internationally, concerns have been voiced at governmental level and by scientists regarding possible adverse health outcomes from frequent wireless phone use by young people [8]. Cellphones are equipped with Adaptive Power Control (APC), which reduces the power output to the minimum necessary to establish a good connection. Cordless phones are a type of cellphone but very few, and none in New Zealand, have APC; they function on full power at all times providing the base is plugged in and turned on at the wall.

Potential vulnerability to neurological and other health effects from exposure to radiofrequencies and extremely low frequencies is commonly regarded as higher in young people than adults [9]. Discussions among the scientific community now seek the best methods for risk management and prevention of harm [10]. Recommendations for a precautionary approach or for children to minimise their use of cellphones are common [8,11]. New Zealand, however, does not recommend reduced use of wireless phone by children, but states that “use of cellphones by children should be a matter for informed choice by parents” [12].

Studies have examined the relationships between duration and intensity of wireless phone use and several types of brain tumour. The most consistently found risks appear to be from intensive use over a few years, extensive use over ten or more years, use predominantly on the side on which the tumour appears (adult studies), and living rurally. There have been only two publications involving people younger than 20 years. One of these [13] found a consistently greater risk for those whose first use of wireless phones was before the age of 20. The other found an exposure-response association between brain tumours and the side of the head next to which the cellphone; these were statistically significant for subscriptions > 4 years (operator recorded data) (Table five) [14]. It was unexpected to find an increased risk for opposite side use, but we note the study did not control for wearing metal-framed eyeglasses. Davias and Griffin explain that the basic resonant frequency for the whole frame of metallic glasses is approximately 900 MHz [15], the same as that on which many cellphones and cordless phones operate. Could this impact on opposite-side RF absorption?

Aydin et al. [14] also reported a statistically significantly increased risk of tumours in brain locations other than temporal, frontal lobes and cerebellum in regular cellphone users, locations where exposure is highest when the phone is held at a normal angle to the head. They argued against a causal relationship.

A few case–control studies have evaluated tumour risk from cordless phone exposure. These have found a statistically significant increase in risk of malignant tumours and benign tumours related to extended hours and years of use [16,17].

Findings have not been consistent across all studies. The most notable problem, common to all, is the large variance of residuals for recalled to billed cellphone use. This is likely due to being asked to recall use from many years ago, further confounded by answers from participants with brain tumours being affected by reduced cognitive acuity. Despite the problems faced in doing case–control studies, they provide the most robust evidence [18].

No studies in the peer-reviewed literature have explored the extent of wireless phone use among New Zealand’s school-age population.

Our aim was to find out how, and to what extent, New Zealand adolescents are typically using and exposed to active cell phones and cordless landline phones (active denotes switched on, transmitting or not, including stand-by), and to consider implications of this in relation to brain tumour risk, with reference to current research findings.

Our focus was on self-reported user-habits. Actual SMS (text) records provided a baseline by which to assess the reliability of self-reporting.

## Methods

### Participants and setting

This cross-sectional survey explored adolescents’ wireless phone user-habits. Sixteen of the 142 schools in the Wellington region of New Zealand each nominated one year 7 and/or 8 class to take part. This amounted to 3% of the region’s year 7/8 population, and provided a representative sample based on school type (years 1–8, year 7–8, years 1–13, and years 7–15) and socio-economic school ratings (decile 1–3, decile 4–7, decile 8–10). Schools are allocated a decile number by the Ministry of Education indicating the proportion of students drawn from low socio-economic communities; the indicator is based on Census data for households with school-aged children in each catchment area [19]. Decile groups are equated here with socio-economic status (SES). The ratio of students at low: mid: high decile schools in this region was approximately 5:10:16. There were 373 participants aged 10.3 - 13.7 years (mean age 12.3), representing an 85% response rate. There were 207 male (55.5%), 165 female (44.2%) and 1 transgender (0.3%) participants. Most were aged 11 or 12 (87.4%) with 83% of the remainder being 13 years old.

The study population was drawn from across New Zealand’s Wellington Region. This includes the capital city, several smaller urban centres, small towns and rural areas.

Participants completed a questionnaire based on that of the MoRPhEUS study (Abramson et al. 2009) and took measurements of phone-to-body distance during use. Working in pairs, participants measured their normal texting distances when sitting and when lying in bed (if used that way). One sat holding the phone as usual and the partner measured the distance to the phone from the abdomen, then (for those who used their phone in bed) the phone holder lay down and held the phone as used in bed while the partner measured the distance from the phone to the bridge of the nose.

### Exposure-metrics

Main exposure-metrics included estimated time spent with an active cell phone close or adjacent to the body, estimated time and number of calls on either phone type, estimated and actual extent of SMS text-messaging (texting), functions used, category of people texted, and use at school. The last of these has been reported elsewhere [20].

### Statistical analysis

Relationships were assessed using Pearson Chi^2^ tests and Pearson’s correlation coefficient (r). A *p* value of 0.05 was considered statistically significant. We applied a method of reducing estimation bias [21] to one recalled phone use variable for comparison. Analyses were undertaken using SPSS version 19.0.

### Ethics

Ethical approval was given by the Victoria University of Wellington human ethics committee. Informed consent was obtained from principals of participating schools and parents of participating students.

## Results

### Cellphone user habits

**Figure 1:**
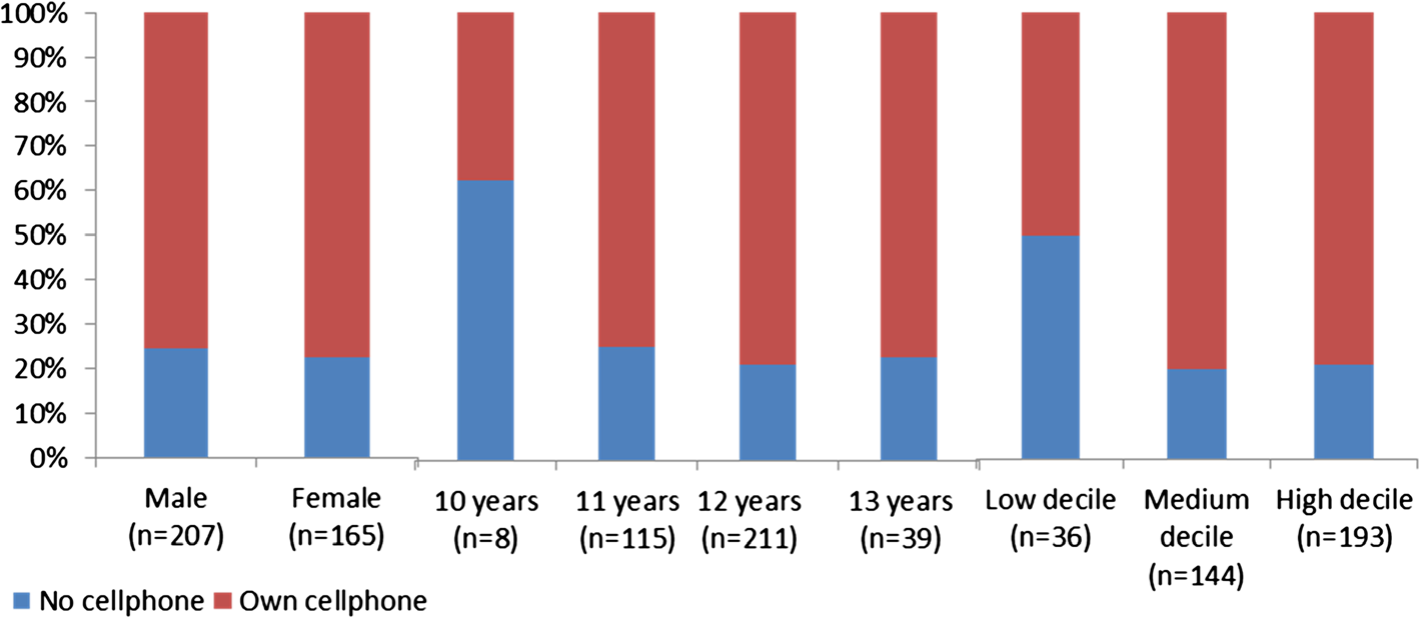
**Short title: Comparative percentage distributions of cellphone ownership by gender, age and school decile (SES).** Legend: Three quarters of students owned a cellphone (70% owned one, 6% owned 2). Cellphone ownership was similar for girls and boys, and there was not a statistically significant difference in ownership by age, although ownership was proportionally lower among 10 year olds. Those in low decile schools (poorer SES) were less likely to own one.

**Figure 2:**
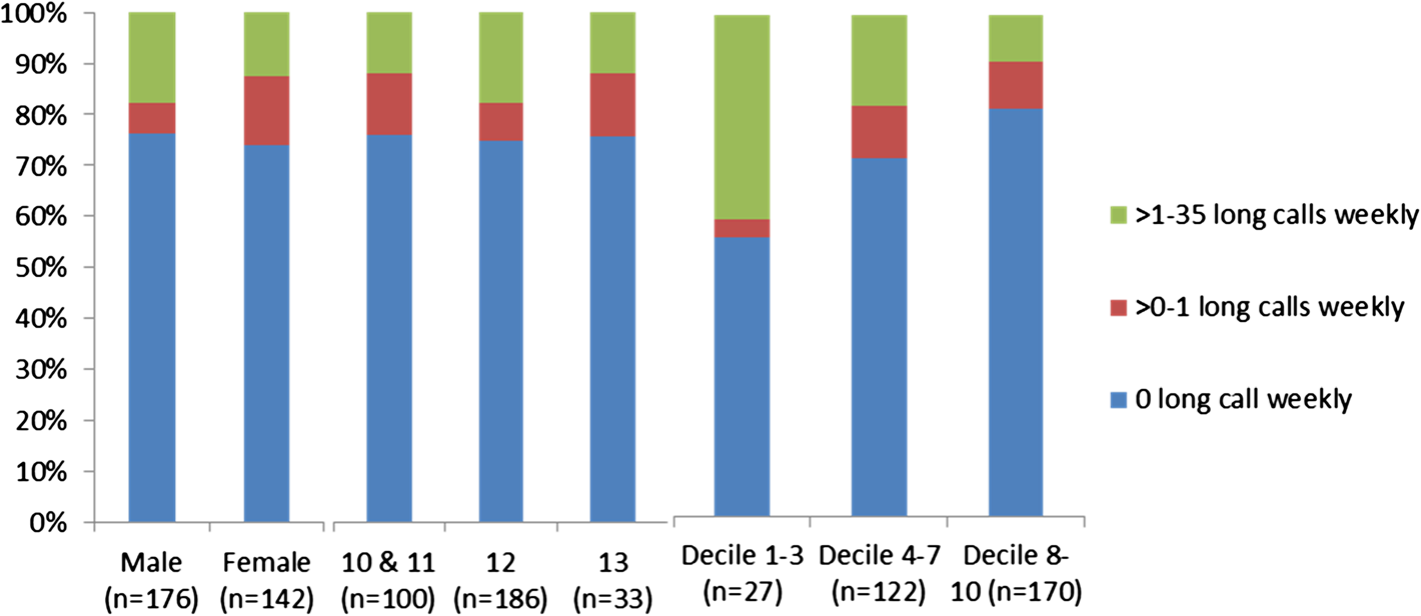
**Short title: Comparative percentage distribution of long cellphone calls (>10 minutes) made and received weekly.** Legend: Use of cellphones for long calls was light. There was no statistical difference according to age or gender, although girls were comparatively less likely than boys to make more than 1 long call weekly. Of those low decile students who made long cellphone calls, they were proportionally more likely to spend extended periods on the cellphone than the high decile students. There were too few 10 year olds to include them as a separate category in the analysis.

Age of first cellphone use peaked at 10 years, but 37% of participants first used one at ages 7 to 9, and 5.5% reported first using one before the age of 7. Years of cellphone use was slightly positively skewed; the median was 2.77 years (interquartile range 2.47). Cellphone ownership at the time of the survey is shown by age, gender and decile group at Figure 1. Most students regularly used a cell phone (70% owned one, 6% owned two, 12.5% regularly borrowed). There was no clear association between age and long cellphone calls weekly (N=319, χ^2^ 3.34, df4, *p*=0.503). Boys made more long cellphone calls, although this was not statistically significant (N=318, χ^2^ 5.53, df2, *p*=0.063). Percentage distributions of long cellphone calls made and received according to gender, age and school decile group are shown in Figure 2.

Cellphone ownership was influenced by socio-economic factors (N=373, χ^2^ 7.493, df2, *p*=0.0004), with those in low-decile schools less likely to own one. However, many students borrowed cellphones, and SES and cellphone calls were negatively associated (N=319, χ^2^ 19.380, (df4), *p=*0.001), with >1 long call weekly associated with *low* SES (*p=*0.00014).

**Figure 3:**
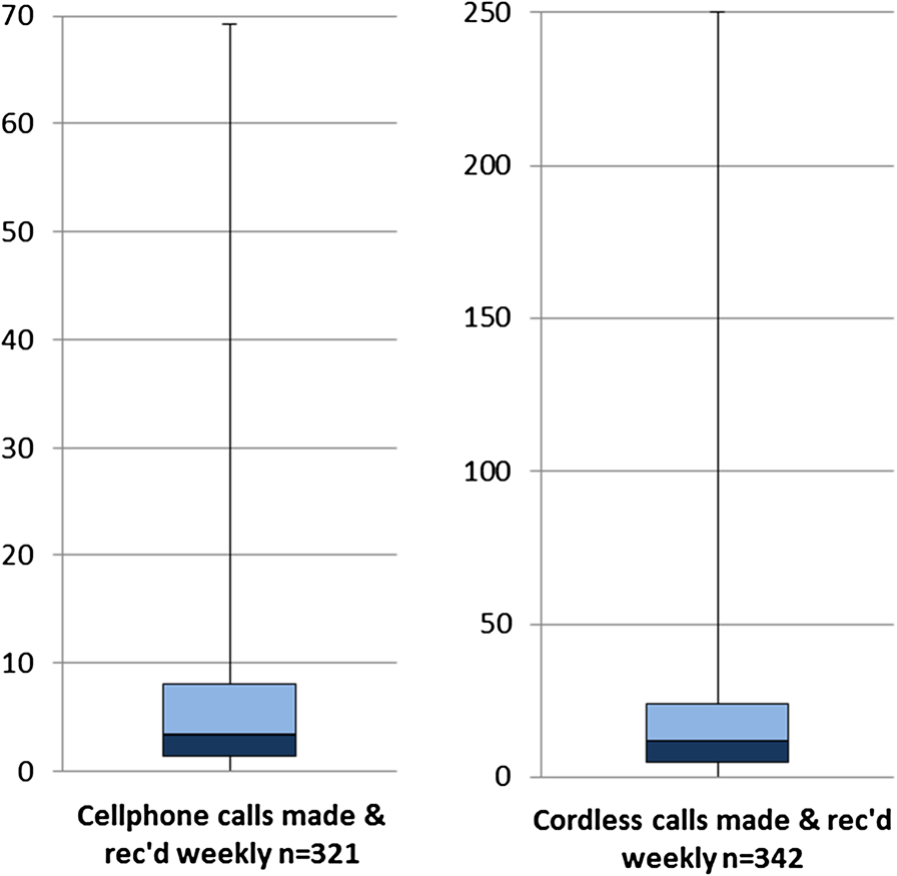
**Short title: Box and whisker plots of total wireless phone calls weekly.** Legend: Cellphone and cordless phone use had similar distributions, but cordless phone use was much greater. The blue boxes indicate the 75^th^ and 25^th^ percentiles and the median.

Reported cell phone use had a positive skew (Figure 3). The median number of weekly voice calls was 3.2 (interquartile range 6.9, full range 0–69). The median number of billed weekly texts was 103 (interquartile range 217, full range 0–1187). Texting, receiving calls and taking photographs were the most popular functions (Table 1) with at least 70% of cell phone owners having a texting plan. Participants could also nominate other functions they used. The most popular self-nominated uses were as an alarm and as a calculator. More than half (58% of cellphone users) reported that they sent most texts to friends. Almost 5% of cellphone users said their most texted person was not a parent, friend or relative (Table 2).

**Table 1:** Cellphone functions used by participants

***Survey categories:***	**% of all participants (N)**	**% of those who used a cellphone (N=331)**
SMS texting	80.7 (301)	90.9
Receiving calls	60.1 (224)	67.7
Camera	59.0 (220)	66.6
Online games/music/internet	46.4 (173)	52.3
Making calls	39.1 (146)	44.1
***Self-nominated categories:***		
Alarm	13.7 (51)	15.4
Calculator	8.8 (33)	10.0
Play stored games	4.6 (17)	5.1
Calendar	4.6 (17)	5.1
Bluetooth	4.0 (15)	4.5
Listen to stored material	3.2 (12)	3.6
Watch	2.7 (10)	3.0
Voice/video recorder	2.4 (9)	2.7
Timer/stopwatch	1.9 (7)	3.1
Send photos	1.9 (7)	2.1
Screen saver/tones etc.	1.9 (7)	2.1
Social networking	< 1% (1)	< 1% (1)
Check account	< 1% (1)	< 1% (1)

Not all functions were available on all phones. It is possible that some participants allocated social networking to ‘internet’ use.

**Table 2:** People most-to-least texted

	**Most texted**	**2**^**nd**^**most texted**	**3**^**rd**^**most texted**	**Least texted**
Friend	**193**	61	24	18
Parent/caregiver	79	**127**	51	24
Other relative	16	55	**129**	71
Someone else	16	37	64	**153**

Not all participants answered all rankings. Equal rankings (n=31) encompassed all combinations and are not shown. Bold print indicates the most prevalent response for each textee’s popularity.

The two most common places that cell phones were carried were a side pocket in trousers or skirt (66%) and a hoodie side pocket (18%). There was a wide variety of locations for carrying a cell phone, a more unusual one being under the bra strap or in the bra which three girls, each from different schools, reported. Cellphones were routinely kept turned on when being carried (90%). Approximately 20% of cell phone owners kept their phone active and in a pocket more than 10 hours daily. The duration of carrying a cell phone by day and having it turned on at night were positively related (χ^2^ 35.96, 3df, *p* <0.00001).

Many sent texts daily from inside the pocket (n=136, 36.6%), and 64.9% (n=242) sent texts with the phone resting in the lap. The median measured distance from the face for normal texting while standing was 30 cm, with 20 cm to the abdomen when lap texting and 23 cm to the eyes when texting in bed. Six students reported usually sending texts with the phone against the abdomen; eighteen usually texted from within 10 cm of the eyes when in bed.

### Cellphones at night

Two-thirds of cellphone owners kept their cellphone beside the bed at night, 12.4% kept it under the pillow. Location of the phone during the day was related to that at night (χ^2^14.5, 4df, *p* = 0.006) with a positive association between keeping it in a pocket by day and under the pillow at night. Having the phone in or beside the bed was positively associated with it being switched on overnight (χ^2^ 11.46, 2df, *p* < 0.003). More than a third (37%) of those who kept a cellphone beside or in their bed at night reported being woken by it at least weekly; having an active phone nearby overnight was related to being woken at least once a week (χ^2^ 53.4, 1df, *p* = <0.00001). One third reported being woken regularly by their phone (13% 1–2 times weekly, 10% 3–4 times weekly, 7% 5–10 times weekly; 3% 11—100 times weekly). Being woken at night was reflected in being chronically tired at school (χ^2^ 16.8, 1df, *p* = 0.00004).

### Cordless landline user-habits

**Figure 4:**
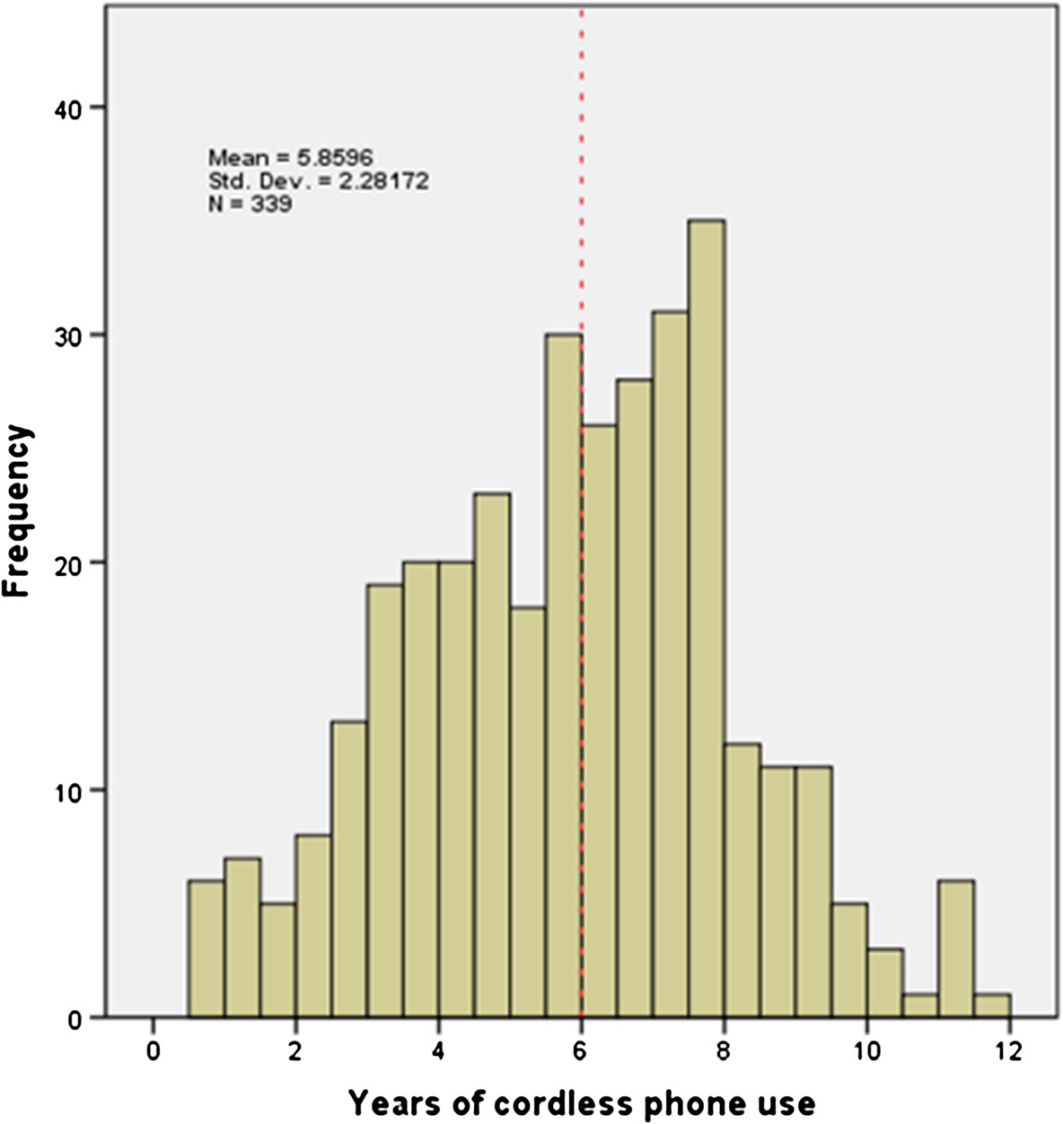
**Short title: Years of cordless phone use at the time of the survey (mid 2009).** Legend: The mean age participants reported starting cordless phone use was 6 ½ years. All bars to the right of the broken line indicate students who will have had ≥ 10 years’ use by mid-2013.

Most (N=341, 91.4%) participants reported using a cordless phone at home. The mean reported period of cordless phone use was 5.9 years (student data) (Figure 4), and the mean period of cordless phone ownership 8.3 years (parent data). Almost one third (n=117) had used a cordless phone for ≥ 7 years. Socioeconomic influence was apparent regarding the type of cordless phone at home (N=127, χ^2^ 12.727, df2, *p=*0.002), with those in the highest SES group being more likely to own a newer model Digital Spread Spectrum Frequency Hopping (DSS FH) cordless phone, while low SES group was associated with not having one at all.

The number of calls made and received weekly on a cordless phone was positively skewed and had a median of 11.8 (interquartile range 19.0, full range 0–250) (Figure 3).

**Figure 5:**
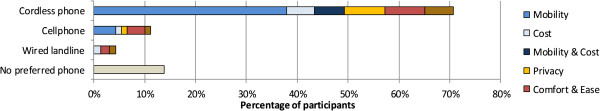
**Short title: Preferred phone for long calls made at home, with reasons.** Legend: Cordless phones were by far the most popular for long calls from home. Participants provided reasons for their choice of favourite phone from which the categories shown in the legend were compiled. Mobility was the most important reason. For many, this was to allow them to do something else at the same time. N=369.

The cordless landline was by far the most popular phone for long calls from home (70%), while 11% preferred a cellphone and fewer than 5% a wired landline (Figure 5). The price structure for landline calls in New Zealand means that local calls are essentially free, being included in a fixed monthly line rental. There was no association between SES and the time spent daily on a cordless phone (N=324, χ^2^ 4.23, df6, *p*=0.645).

**Figure 6:**
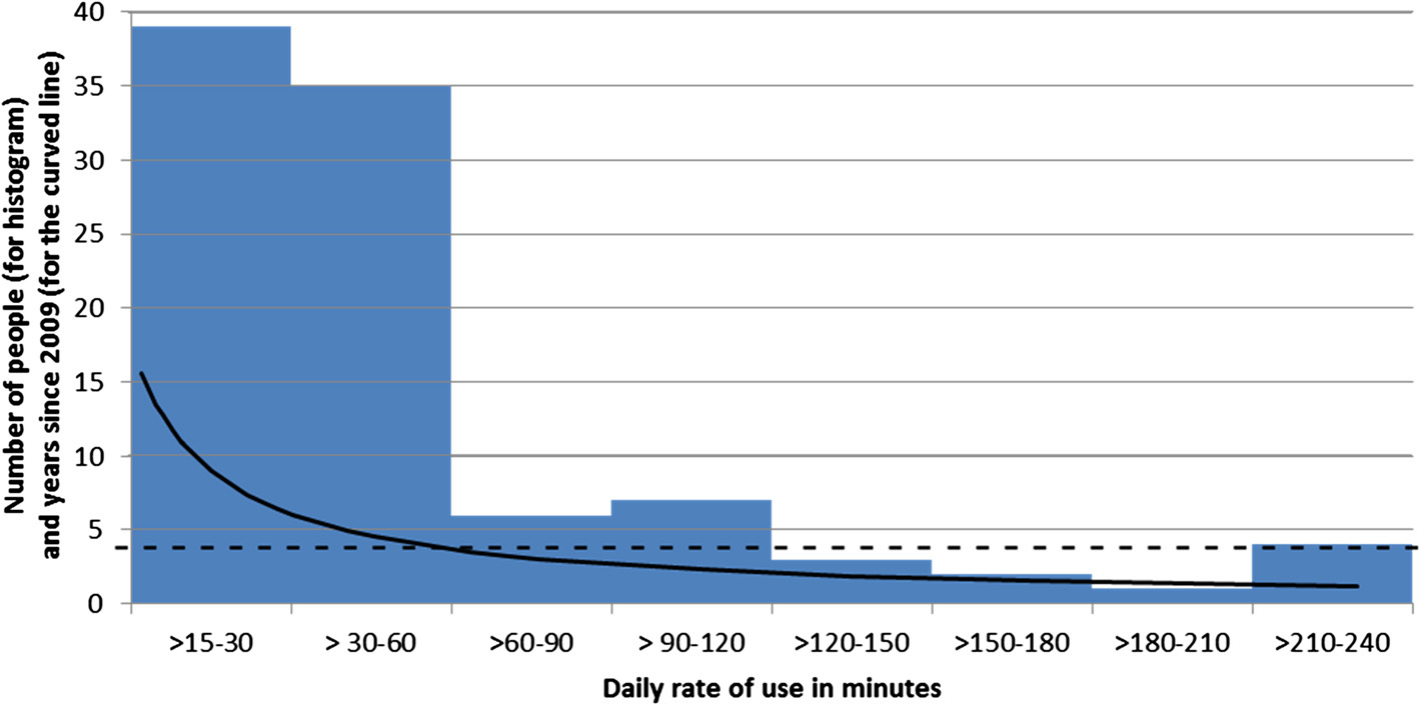
**Short title: Time since survey to reach 1640 hours’ cordless phone use at the reported daily rate.** Legend: This figure has a dual purpose. It shows both the reported time spent daily on a cordless phone (as a histogram) and the years since 2009 that it will take for participants to reach 1640 hours on a cordless phone at the reported rate of use (the black curved line). The curved line charts the critical rate of use over x years to reach 1640 hours’ use since the survey. The broken line indicates mid-2013 (4 years since the survey). All those to the right of where the lines cross (i.e. more than 60 mins/day) will have had ≥ 1640 hours’ exposure by mid-2013. This is equivalent to the top decile Interphone use. Previous use and cellphone use are not included. Only those who reported >15 minutes/day are shown in the histogram.

**Figure 7:**
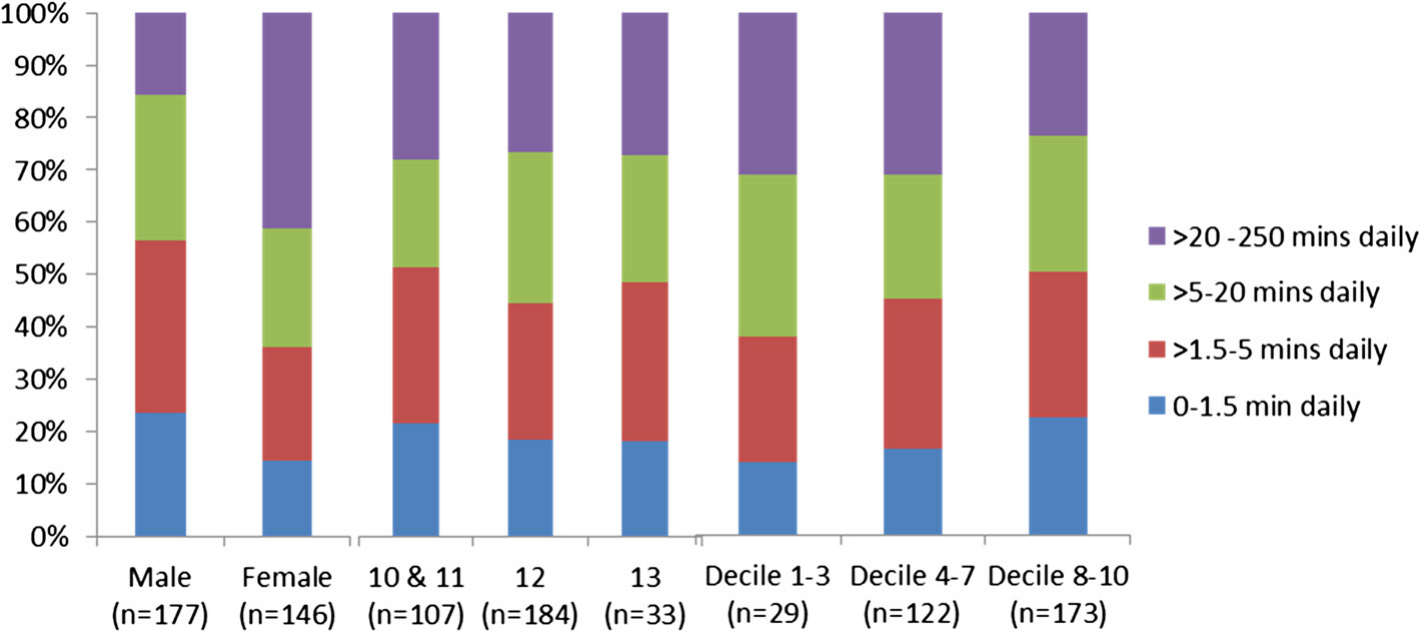
**Short title: Comparative percentage distributions of daily minutes spent on a cordless phone.** Legend: Cordless phone use did not differ by age or school decile (SES), but girls were more likely to spend extended periods on the cordless phone. There were too few 10 year olds to include them as a separate category in the analysis.

Recalled time per evening on a cordless phone also had a strong positive skew (histogram component of Figure 6). Students were asked how long they spent daily, on average, on the cordless phone between the end of school and when they went to sleep. Some reported in minutes and some in fractions of hours. The median time was 5 minutes. However, a third of cordless phone users (32.8%) reported spending ≥ 15 minutes per day on one and 23.8% spent 30 minutes per day on one. Applying a method [21] to reduce estimation bias of daily minutes on a cordless phone reduced original estimates that were > 60 and increased those that were < 60. The resulting forecast values suggested almost half those with a cordless phone (47.6%) spent ≥ 15 minutes on a cordless phone daily and 25.3% spent ≥ 30 minutes. Girls were statistically significantly more likely than boys to spend a longer time on a cordless phone daily (N=323, χ^2^ 26.54 (df3) *p*<0.00001). There was no clear association between age and cordless phone minutes daily (N=324, χ^2^ 2.66 (df6) *p=*0.850) (Figure 7).

Cellphone and cordless phone use were correlated (Pearson r 0.255, 2-tailed, *p*<0.0001).

Parents’ perception of possible health risks from wireless phones was greater for cellphones than cordless phones (Table 3).

**Table 3:** Parents’ perception of health risk from wireless phones

**Risk concern**	**Cellphone % (N=325)**	**Cordless phone % (N=324)**
None	7.4	23.5
Low	37.2	34.9
Moderate	29.8	11.1
High	7.1	4.0
Don’t know	18.5	26.6

Parents’ perception of health risk was greater for cellphones than cordless phones.

## Discussion

The use of both cellphones and cordless phones was a normal activity for the large majority of participants and each was used very differently although the amount of cordless phone use was positively and systematically related to the amount of cellphone use, as reported elsewhere [6,22]. The cellphone was more popular for texting, for internet, games and music, as a camera, and for receiving phone calls than for making calls. The cordless phone was clearly the most popular choice for long phone calls.

We now discuss some long-term health considerations. In the years since the study, the popularity of the most popular functions described above has grown, with social networking increasing in popularity among adolescents since 2009 [1]. In our study, many students used the phone in their lap, sometimes with the lower edge resting against the abdomen with the phone at right-angles to the body. Peak-penetration of the energy is focused more deeply when the phone’s antenna is at right angles to the body [23]. A majority carried it in a pocket, with many texting from that location. Smartphones, which have rapidly gained popularity with adolescents [24,25] present new challenges as they are continually transmitting data, especially when connected to Twitter or Facebook, and continue to do so while in the pocket (personal communication, C Zombolas, Managing Director, EMC Technologies, 5 December 2012). Use of a cellphone in these locations could be of concern for future fertility [20].

Another consideration is the growth plate of the femur, located in the metaphyseal region, which would lie directly under the side pocket in many cases. The femur and growth plate are in a highly proliferative state during the adolescent years.

Exposure of T-lymphoblastoid leukaemia cells to unmodulated 900 MHz frequencies has been demonstrated to increase apoptosis (natural cell death), but continued exposure resulted in pro-survival signals preventing death of damaged cells [26]. Other research observed increased inhibition of DNA repair foci in stem cells after exposure to typical GSM and UMTS signals; the effect was thought to be caused by the extremely low frequencies resulting from modulation [27]. Fibroblasts mostly adapted when exposure was chronic, but stem cells did not.

Increased protein synthesis has been observed when proliferating human fibroblasts were exposed to low intensity 1800 MHz radiofrequency [28], commonly used by cellphones as a carrier frequency.

There has so far been no research examining bone cancer and cellphone radiofrequency exposure, although there are a few leukaemia studies. In vivo research of radiofrequency impacts on fertility parameters has been restricted to human adults and animals.

### Implications for brain tumour risk

Cardis and Sadetzki, lead researchers in the Interphone study, remark that, “Indications of an increased risk in high- and long-term users from Interphone and other studies are of concern” (p.170) as, “Even a small risk at the individual level could eventually result in a considerable number of tumours and become an important public-health issue” [29]. It is, then, appropriate to compare our young generation’s extent of phone use with that which has been found in various studies to be related to increased risk of brain tumour.

The distribution of calls made and received on these phones was very similar, but the extent of cordless phone use was much the greater and their years of use were longer. This means that overall radiofrequency exposure in the brain of the participants was likely to be greater from their cordless phone use than cellphone use. By mid-2013, 46% of all participants will have used a cordless phone for ≥ 10 years.

In 2010, the International Agency for Research on Cancer met to assess the carcinogenicity of RF. After evaluating the available research, the committee rated “radiofrequency electromagnetic fields, such as, but not limited to, those associated with wireless phones” as a 2B carcinogen. This decision largely hung on the evidence presented in two large case–control studies: a pooled analysis of 2 case–control studies of wireless phone use and the risk of malignant brain tumours by the Hardell-group [30] and the 13-country Interphone study [31].

Most of the Interphone results were statistically insignificant or even suggested either a protective effect or methodological problems, but there were a few statistically significant results in categories of heaviest or longest use [31]. An association of intensive and extended wireless phone use with some brain tumours is common to most studies in this area.

One studied tumour-type has been gliomas, which are generally malignant. Interphone participants had an odds ratio (OR) 1.40, 95% confidence interval (CI) 1.03-1.89, between ≥ 1640 hours cellphone use and glioma, while that extent of use over only 1–4 years before the reference date had an OR 3.77, 95% CI 1.25-11.4 [31]. Intensity of use appears important as, when only those with ≥ 10 years use were considered, the result was not statistically significant (OR 1.34, 95% CI 0.90-2.01). Odds ratios were higher when proxy interviewers were excluded and only data collected by experienced interviewers used.

In the Hardell-group pooled analysis, the highest OR was in those who began wireless phone use before the age of 20 years and had >1 year’s use [16]. The odds ratio of malignant tumour for this age group from cordless phone use was OR 2.1, 95% CI 0.97-4.6 while for digital cellphones it was OR 3.7, 95% CI 1.5-9.1. When data for those with >1 year’s wireless phone use (all age groups) were analysed, neither cellphone nor cordless phone use were independently related to increased malignant tumour incidence [16]. But all *combinations* of phone use were. For instance, use of both a digital cellphone and a cordless phone had OR 1.4, 95% CI 1.1-1.8 while analogue cellphone and a cordless phone had OR 1.6, 95% CI 1.2-2.2. In our study, 274 participants (74%) had used both a cordless and cellphone for more than a year.

In many respects, the Interphone findings were not consistent with those found in other studies, particularly those of Hardell’s group. These differences have been analysed [32,33], and the authors point out that Hardell’s studies generally include a higher number of participants with ≥ 10 years’ use. The methodology of the two groups also differed. When Hardell’s group re-analysed their case–control glioma study [16] using the same criteria as that in the Interphone study Appendix 2 [34] the results for ≥ 10 years and cumulative use ≥ 1640 hours were similar [35]. For instance, the ORs and 95% CI for those with glioma and ≥ 1640 hours use were 1.89 (1.08-3.30) compared to 1.82 (1.15-2.89), respectively. This represented those aged 30–59. Further analysis by the Hardell-group study, including ages 20–59, increased the OR to 2.23 (1.30-3.82).

If the reported rate among those in our study using a cordless phone stayed the same since participating in the survey, and if cordless phone and cellphone use carry a similar risk, the total hours of intensive cordless phone use alone will place 22 students in our study (6%) in the category of at least 1640 hours’ use over the 4 years from the survey to mid-2013, suggesting a 3.77-fold increased risk of glioma. At that time, their average age will be 16¼ years.

The present study used participants’ self-reported data. Four factors suggest 1640 hours of use would be reached sooner rather than later by the heavy users. Firstly, prior cordless and all cellphone use are not included in calculations of the time it will take to reach 1640 hours’ use. Secondly, the extent of cordless phone use is positively related to that of cellphone use for calls, both in this study and elsewhere [6,22], so heavy use of one phone type is compounded by heavy use of the other. Thirdly, several studies have shown that adolescent wireless phone use tends to increase rather than decrease or remain static from pre-adolescence through the high school years [6], not beginning to decrease until the age of 18 [36]. Finally, the heaviest users’ underestimated their extent of texting [37] so this may well also have applied to their estimates of phone use.

## Conclusions

By 2009, New Zealand’s adolescents were using both cellphones and cordless phones extensively and in many ways. The extent and duration of cordless phone use by some students raises concerns that by the age of 16 many were already in a category of increased risk of brain tumours; in the adolescent years leading up to this, their brains are undergoing dramatic transformation [38]. Rodier suggests that because the central nervous system and its myelinisation developmental processes are vulnerable to interference by agents that adult physiology can cope with, it is reasonable to expect that the later stages of brain development present particular risks [39].

The common habit of carrying and using a cellphone in a pocket or the lap suggests a possible avenue for research considering whether radiofrequency exposure from wireless technology is related to tumours found in the proximal femur or pelvis. Important examples are Ewing’s sarcoma, osteosarcoma, and fibrosarcoma of bone, all of which occur most often in young people [40].

New Zealand’s wired landline billing system varies from that of some countries making local cordless calls free while cellphone calls are relatively costly. So, while the balance of cordless to cellphone use may vary between countries there are common threads. Texting has become popular internationally among young people, and extensive calls on one phone type or another are common among a proportion of that population. Advice to reduce exposure is not likely to be very effective with adolescents who feel impervious to risk. An alternative approach which would enable informed choice is educating children, parents and teachers about radiofrequency technology and the circumstances under which cellphones increase and decrease their energy output. Teens can then be encouraged to formulate ways they can continue using phones while reducing their radiofrequency exposure. Education is a step supported elsewhere [8,10].

## Abbreviations

SMS: Short message service; APC: Adaptive Power Control; OR: Odds ratio; CI: Confidence interval; ICNIRP: International Commission on Non-Ionizing Radiation Protection; SAR: Specific Absorption Rate.

## Competing interests

The author declares that she has no competing interests.

## Author’s contribution

The named author adapted and developed the questionnaire; collected, entered and analysed the data; reviewed the relevant literature; and prepared the manuscript.
